# MRI-Based Radiomics Approach Predicts Tumor Recurrence in ER + /HER2 − Early Breast Cancer Patients

**DOI:** 10.1007/s10278-023-00781-5

**Published:** 2023-01-25

**Authors:** Piero Chiacchiaretta, Domenico Mastrodicasa, Antonio Maria Chiarelli, Riccardo Luberti, Pierpaolo Croce, Mario Sguera, Concetta Torrione, Camilla Marinelli, Chiara Marchetti, Angelucci Domenico, Giulio Cocco, Angela Di Credico, Alessandro Russo, Claudia D’Eramo, Antonio Corvino, Marco Colasurdo, Stefano L. Sensi, Marzia Muzi, Massimo Caulo, Andrea Delli Pizzi

**Affiliations:** 1 Advanced Computing Core, Center of Advanced Studies and Technology (CAST), “G. d’Annunzio” University of Chieti-Pescara, Chieti, Italy; 2 Department of Innovative Technologies in Medicine and Odonoiatry, “G. d’Annunzio” University, Chieti, Italy; 3 Department of Radiology, Stanford University School of Medicine, Stanford, CA USA; 4 Department of Neuroscience, Imaging and Clinical Sciences, “G. d’Annunzio” University, Chieti, Italy; 5 Unit of Radiology, “Santissima Annunziata” Hospital, Chieti, Italy; 6 Unit of Pathology, Breast Center EUSOMA, Ortona, Chieti, Italy; 7 Unit of Ultrasound in Internal Medicine, Department of Medicine and Science of Aging, “G. D’Annunzio” University, Chieti, Italy; 8 Unit of Radiology, “Renzetti” Hospital, Lanciano, Italy; 9 Breast Unit, “Gaetano Bernabeo” Hospital, Ortona, Italy; 10 Motor Science and Wellness Department, University of Naples “Parthenope”, 80133 Naples, Italy

**Keywords:** Artificial intelligence, Breast cancer, Oncotype DX, Machine learning, Magnetic resonance imaging

## Abstract

Oncotype Dx Recurrence Score (RS) has been validated in patients with ER + /HER2 − invasive breast carcinoma to estimate patient risk of recurrence and guide the use of adjuvant chemotherapy. We investigated the role of MRI-based radiomics features extracted from the tumor and the peritumoral tissues to predict the risk of tumor recurrence. A total of 62 patients with biopsy-proved ER + /HER2 − breast cancer who underwent pre-treatment MRI and Oncotype Dx were included. An RS > 25 was considered discriminant between low-intermediate and high risk of tumor recurrence. Two readers segmented each tumor. Radiomics features were extracted from the tumor and the peritumoral tissues. Partial least square (PLS) regression was used as the multivariate machine learning algorithm. PLS β-weights of radiomics features included the 5% features with the largest β-weights in magnitude (top 5%). Leave-one-out nested cross-validation (nCV) was used to achieve hyperparameter optimization and evaluate the generalizable performance of the procedure. The diagnostic performance of the radiomics model was assessed through receiver operating characteristic (ROC) analysis. A null hypothesis probability threshold of 5% was chosen (*p* < 0.05). The exploratory analysis for the complete dataset revealed an average absolute correlation among features of 0.51. The nCV framework delivered an AUC of 0.76 (*p* = 1.1∙10^−3^). When combining “early” and “peak” DCE images of only T or TST, a tendency toward statistical significance was obtained for TST with an AUC of 0.61 (*p* = 0.05). The 47 features included in the top 5% were balanced between T and TST (23 and 24, respectively). Moreover, 33/47 (70%) were texture-related, and 25/47 (53%) were derived from high-resolution images (1 mm). A radiomics-based machine learning approach shows the potential to accurately predict the recurrence risk in early ER + /HER2 − breast cancer patients.

## Introduction

Breast cancer is a leading cause of death and the most common cancer in women [1]. It consists of four main subtypes, classified by tumor genotype and molecular characterization in luminal A, luminal B, HER2-enriched, and basal-like cancer [1–3]. Luminal tumors represent the most invasive breast cancer (70%) in western countries. They are usually estrogen-receptor (ER) and/or progesterone-receptor (PR) positive and HER2-receptor (HER2) negative.

Hormone therapy represents a mainstay for patient management [4]. About 15% of luminal B cancers will develop a recurrence within 10 years from the diagnosis if treated with hormonal therapy alone. Although the risk of recurrence could be lowered by adjuvant chemotherapy, selecting patients who might benefit from adjuvant chemotherapy is debated [5–7].

Oncotype Dx Recurrence Score (RS) 21-gene expression assay (Genomic Health Inc., Redwood City, CA) produces a score based on the quantitative expression level of 21 genes in ribonucleic acid extracted from formalin-fixed, paraffin-embedded breast tumor tissues. This assay has been validated in patients with ER + /HER2 − invasive breast carcinoma to estimate patient risk of distant breast cancer recurrence and guide the use of adjuvant chemotherapy [8–11]. Recent studies demonstrated that RS correlates with recurrence rates and adjuvant treatment response [5, 9, 12–14]. The National Comprehensive Cancer Network (NCCN) and the American Society of Clinical Oncology (ASCO) both recommended the use of Oncotype Dx RS testing in patients with ER + /HER2 − breast cancers [15, 16]. Moreover, the Guidelines Development Group of the European Commission Initiative on Breast Cancer recently prioritized a clinical question on the use of multigene test to guide the use of adjuvant chemotherapy in ER + , HER2 − , and lymph node–negative or up to 3 lymph node–positive invasive breast cancer [17]. For these reasons, this assay is now incorporated into clinical practice guidelines for treatment decisions [12]. However, the technique is costly and is performed on surgical breast tumor specimen. These limitations prompted researchers to investigate new imaging-based biomarkers [18–24]. Multiparametric magnetic resonance imaging (MRI) is the most sensitive modality to diagnose and assess treatment response in breast cancer patients [25–28]. In this regard, recently developed imaging-based methods, such as radiomics, allow analyzing imaging data and extracting many quantitative features, thereby adding a whole tumor volume of extra information to the conventional qualitative visual assessment [18–24, 29–35]. MRI-based predictors of tumor recurrence allow the non-invasive selection of patients at high risk of recurrence, with significant improvements on patient healthcare and overall costs. Few studies investigated the use of MRI-based radiomics for the prediction of breast cancer recurrence having the Oncotype Dx RS as reference standard [36–44]. Compared to previous studies on Oncotype Dx, ours not only was focused on the tumor but also investigated the peritumoral tissues. In fact, as in other recent studies, not only on breast cancer, radiomics features extracted from the tumor site and the peritumoral environment showed a potential role in terms of prediction of treatment response [45–48]. Moreover, the American Society of Clinical Oncology (ASCO) recently revised the breast cancer recurrence risk by addressing the use of Oncotype Dx in guiding decisions on the use of adjuvant systemic therapy. In detail, they divided high risk and low-intermedium risk based on a RS cut-off of 25 [9]. In this regard, only one study was recently published on the potential correlation between radiomics and RS adopting this cut-off, but it did not assess the role of peritumoral tissues [49].

This study investigated the ability of MRI-based radiomics features extracted from the tumor and the peritumoral tissues to predict the risk of tumor recurrence in ER + /HER2 − breast cancer patients. Thus, by demonstrating the presence of imaging-based biomarkers, we could non-invasively identify patients who are more likely to benefit from adjuvant therapy.

## Materials and Methods

### Subjects

This study received formal approval from the Ethical Committee of the University G. d’Annunzio of Chieti-Pescara, Italy; informed consent was waived by the same ethics committee that approved the study (Comitato Etico per la Ricerca Biomedica delle Province di Chieti e Pescara e dell’Università degli Studi “G. d’Annunzio” di Chieti e Pescara). The study was conducted according to ethical principles laid down by the latest version of the Declaration of Helsinki. A total of 62 patients who underwent clinically indicated breast MRI between January 2016 and May 2020 at our institution were retrospectively included. Inclusion criteria were as follows: [1] ER + /HER2 − early breast cancer confirmed via biopsy, [2] MRI performed on a 1.5-T scanner, and [3] availability of Oncotype DX RS.

### MRI Protocol

All patients in this cohort underwent a clinically indicated breast MRI consisting of a standard T1-weighted (T1w), T2-weighted (T2w), diffusion-weighted imaging (DWI), and dynamic contrast enhancement (DCE) acquisition performed using a 1.5-T MR scanner (Achieva, Philips Medical System, Best, the Netherlands) equipped with a dedicated phased-array breast coil. Detailed information regarding the DCE acquisition is described in Table 1.

**Table 1:** MRI protocol parameters

	T1-weighted post-contrast 3D-FFE
Repetition time (msec)	3000–5000
Echo time (msec)	80
Section thickness (mm)	2
Section gap (mm)	0
Acquisition matrix size	340 × 340
No. of signals acquired	2
Field of view (mm)	340 × 340
Sensitivity encoding (SENSE)	Yes
Acquisition time (sec)	54.3, 90^**^
No. of sections	167

*FFE*, Fast Field Echo

^**^First (“early”) and second (“peak”) DCE acquisition after the endovenous administration of contrast agent (gadolinium chelate)

### Imaging Analysis

**Fig. 1:**
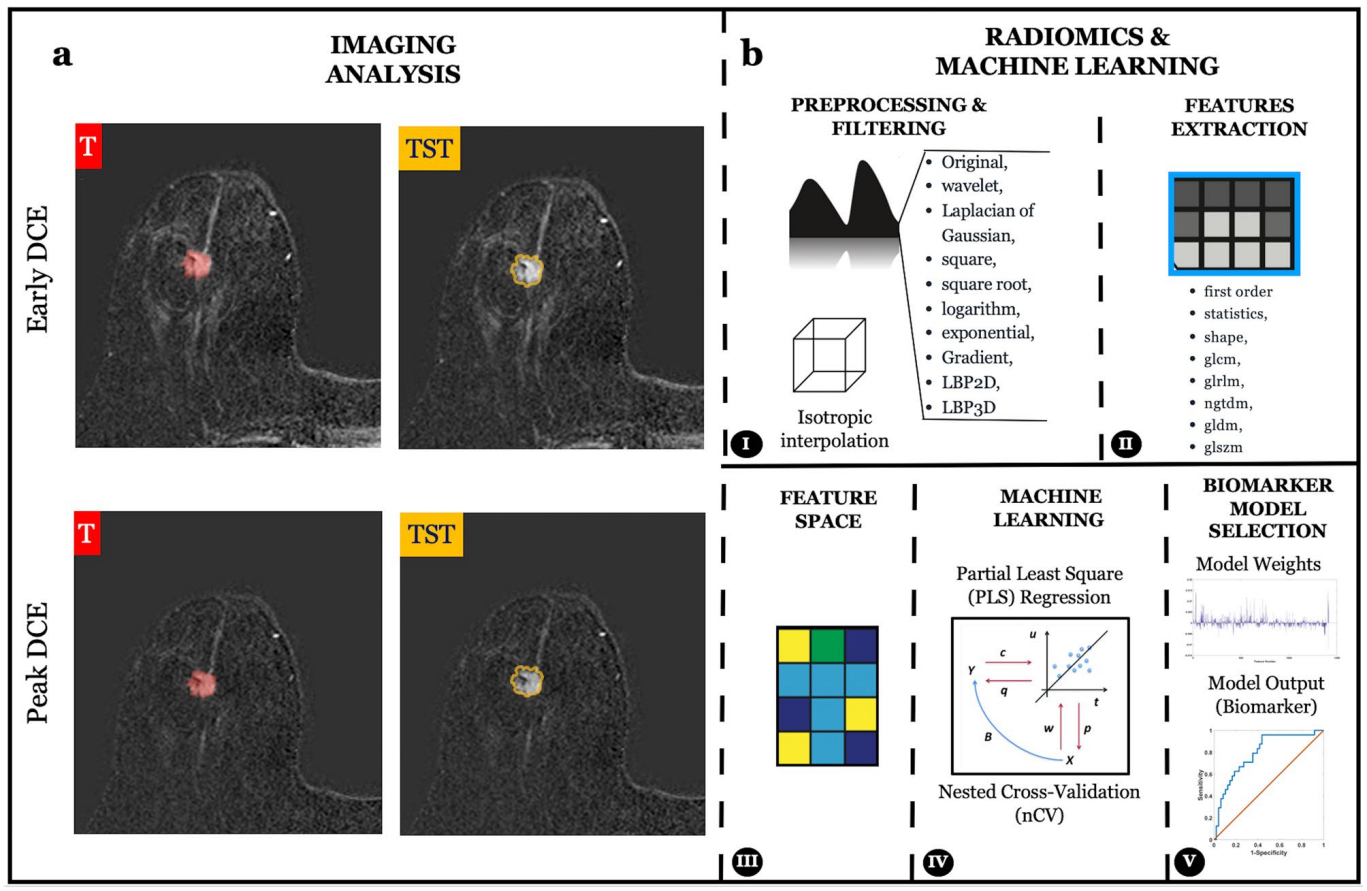
**a** Tumor (T) and tissue surrounding tumor (TST) segmentation on the first (“early”) and second (“peak”) contrast-enhanced dynamic (DCE) T1w images. **b** Schematic representation of the radiomics features extraction and the machine learning framework implemented

**Fig. 2:**
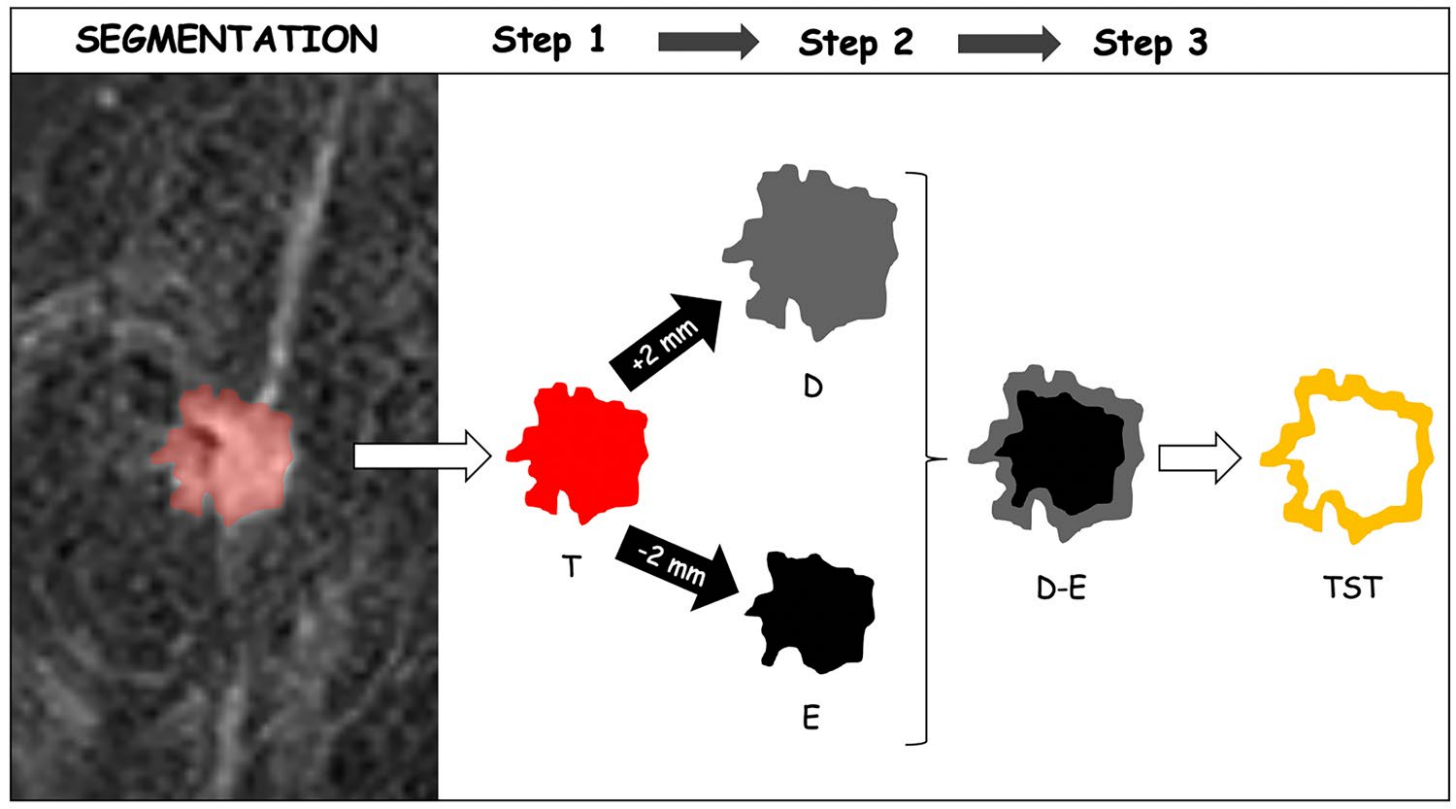
The segmentation process included three steps. Firstly, the whole breast tumor (T) was manually segmented on contrast-enhanced dynamic T1w images. In the second step, the edge of T was dilated (D) and eroded (E) by 2 mm, respectively. In the third step, we overlapped the dilated and eroded masks and subsequently subtracted them to include the most peripheral portion of the tumor and the surrounding tissues (TST)

Whole volume tumor manual segmentation of the tumor (T) was performed on the first (“early”) and second (“peak”) contrast-enhanced dynamic T1w images for each patient by two independent senior radiology residents. The software used for the segmentation was an open source medical image computing platform, 3D Slicer Version 4.8 (www.3dslicer.org). To create the “tissue surrounding tumor” segmentations (TST), a “3dmask_tool” (AFNI) was used [50]. First, a 2-mm dilatation (“dilate”) and a 2-mm erosion (“erode”) were obtained from the CT of each patient. Then, the two masks were subtracted (“dilate” − “erode”) to obtain the TST which was 4 mm thick (Fig. 2) [45]. All the TST segmentations were then checked by the two readers and manually adjusted if necessary to include only the outer border of the tumor and the adjacent perivisceral tissue. T and TST are shown in Fig. 1a.

### Radiomic Features Extraction

The extraction of radiomics features from the masked (T and TST) T1w images was performed using PyRadiomics [51]. Reproducibility assessments of the features extracted by the two readers from the segmentations of all patients were performed (Fig. 2). To avoid data heterogeneity bias and minimize acquisition-related radiomics variability, MR images and masks were resampled using 3 isotropic voxel dimensions (1 × 1 × 1 mm, 2 × 2 × 2 mm, and 3 × 3 × 3 mm). For each segmentation and for each image resolution (1 mm, 2 mm, and 3 mm), ten built-in filters (Original, wavelet, Laplacian of Gaussian (LoG), square, square root, logarithm, exponential, Gradient, LBP2D, and LBP3D) were applied, and seven feature classes (first-order statistics, shape descriptors, glcm, glrlm, ngtdm, gldm, and glszm) were calculated, which resulted in a total of 1409 radiomics features for each image (Fig. 1b) [52–54]. Prior to the machine learning analysis, all features were converted into *z*-scores relying on their subject distribution.

### Machine Learning Analysis

A machine learning approach was used to exploit the radiomics features’ multidimensionality and infer the risk of recurrence (high vs. low-intermedium). Two main strategies were implemented to address the large number of features extracted [55, 56]. The first approach reduced the number of used features by selecting only highly repeatable features between the masks delineated by the two radiologists (*r* > 0.95). The second approach leveraged the high collinearity among radiomics features which was evaluated through an initial exploratory analysis. It then used a linear regression analysis to infer the risk of recurrence, thus employing a space dimension reduction procedure, namely, the partial least square (PLS) regression [55–58]. PLS has one hyperparameter, namely, the number of uncorrelated components to be used in the regression. Leave-one-out nested cross-validation (nCV) was used to achieve hyperparameter optimization and evaluate the generalizable performance of the procedure [58–60]. In nCV, data are divided into folds, and the model is trained on all data except one-fold in an iterative, nested manner. Whereas the outer loop estimates the model’s performances among iterations (test), the inner loop evaluates the optimal hyperparameter (validation). If the number of folds equals the number of samples (one-fold per sample), the procedure is defined as leave-one-out nCV, an approach highly suited for medical applications where samples represent subjects [61–63]. The whole leave-one-out nCV PLS analysis was repeated multiple times for the following group of masks: (a) DCE images (“early” and “peak”) in both T and TST, (b) “peak” DCE in both T and TST, (c) “early” DCE in both T and TST, (d) “peak” DCE in T, (e) “peak” DCE in TST, (f) “early” DCE in T, and (g) “early” DCE in TST.

### Reference Standard

A recurrence score > 25 was considered to discriminate between low-intermediate (≤ 25) and high risk ( >) of tumor recurrence [9, 11, 64, 65].

## Calculation

The classification performances were assessed through receiver operating characteristic (ROC) analyses considering the inferred (out-of-training-sample) recurrence risk in the outer loop fold of the machine learning framework. Patients with low-intermediate recurrence risk were attributed to the “negative” group, whereas patients with high recurrence risk were attributed to the “positive” group. The ROC analyses were also performed on random shuffled outcomes to simulate the null hypothesis and evaluate its confidence interval (repeated 10^6^ times). The ROC analysis delivered an area under the curve (AUC), which, using the random shuffled outcomes, could be transformed into a *z*-score for assessing its statistical significance. The statistical analysis was performed in MATLAB.

## Results

**Fig. 3:**
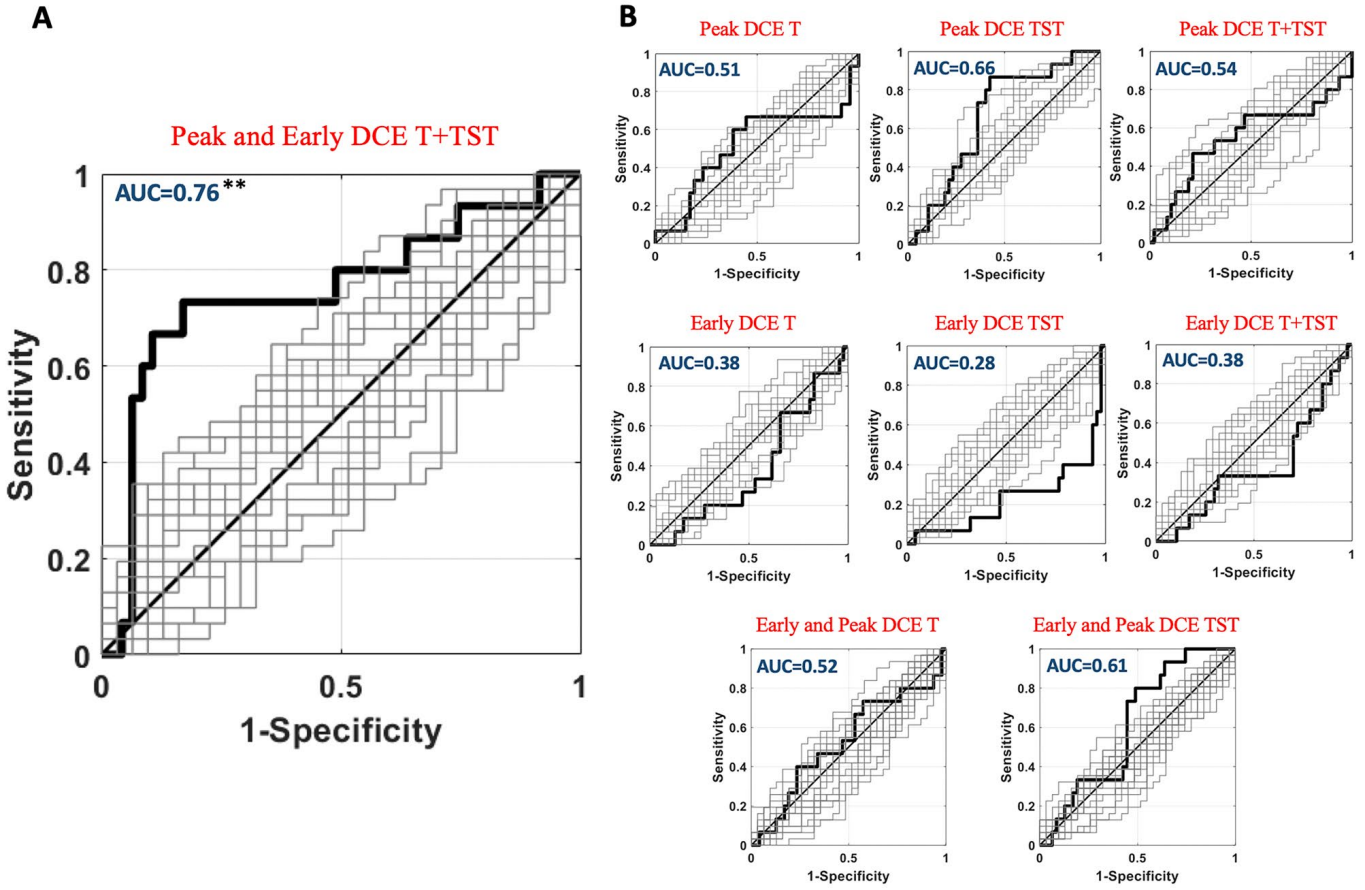
ROC analysis of the machine learning (PLS) classification performance. Patients with RS ≤ 25 were attributed to the “negative” group, whereas patients with RS > 25 were attributed to the “positive” group

**Fig. 4:**
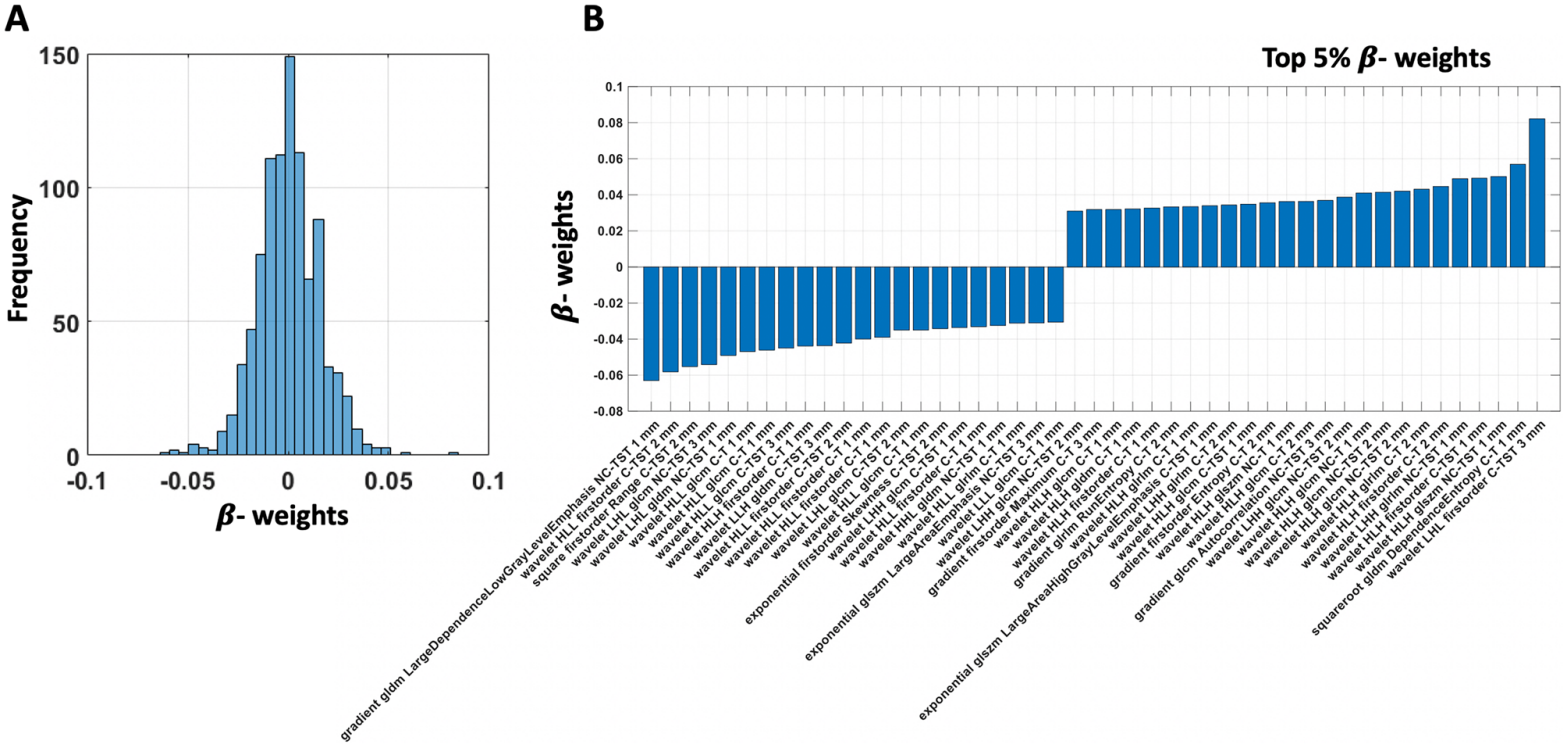
**A** Partial least square analysis showing β-weights associated with reliable (*r* > 0.95) radiomics features. **B** β-weights are associated to the top 5% of features with the largest β-weights in magnitude

Out of the 62 women included in the study, the mean age was 49 (interquartile range: 44.25–53) years. Forty-seven (75.8%) patients showed a RS ≤ 25 (low-intermediate recurrence risk) and 15 (24.2%) showed a RS > 25 (high recurrence risk) (Table 2). In total, 1409 radiomics features were extracted for each image. Each MRI included “early” and “peak” DCE images. We extracted two masks (T and TST) from each set of images (“early” DCE T, “early” DCE TST, “peak” DCE T, and “peak” DCE TST). All MRI images were resampled at 3 resolutions, for a total of 33,816 features per patients. In detail, the number of features selected (based on inter-read repeatability of *r* > 0.95) for each repetition of the analysis was as follows: *n* = 940 from “early” and “peak” DCE T + TST images, *n* = 644 from “peak” DCE T + TST images, *n* = 296 from “early” DCE T + TST images, *n* = 315 from “peak” DCE T, *n* = 329 from “peak” DCE TST, *n* = 230 from “early” DCE T, and *n* = 66 from “early” DCE TST. The exploratory analysis for the complete dataset revealed an average absolute correlation among features of 0.51. The high average absolute correlation among features justified the use of PLS. Using the nCV machine learning PLS framework, a significant inference on the risk of recurrence was obtained when including all features in the analysis (“early” and “peak” DCE T + TST, optimal number of PLS components, *n* = 19), with an *AUC* = 0.76, *z* = 3.01, *p* = 1.1∙10^−3^ (Fig. 3A). Standalone combinations of “early” and “peak” DCE images of T and TST did not deliver a significant multivariate inference of the risk (*p* > 0.05; Fig. 3B). When combining “early” and “peak” DCE images of only T or TST, a tendency toward statistical significance was obtained for TST with an AUC of 0.61 (*p* = 0.05). Figure 4A reports the nCV β-weight distribution depicting the strength and sign of the effect of the original radiomics features in the inference of the outcome. Since the larger labeling value of “1” was associated with an increased risk of tumor recurrence, the positive β-weight suggested a higher risk at increasing feature value and vice-versa for negative weights. Figure 4B reports the top 5% (*n* = 47) β-weights associated with the most relevant features involved in the prediction (those with the largest β-weight magnitudes). These features were balanced between T and TST (23 and 24, respectively). In detail, 25 of the top 5% features were associated with images at 1 mm resolution, 15 at 2 mm resolution, and 7 at 3 mm resolution. Most (33/47) of those features were related to the texture analysis. Thirty-three (70%) top 5% weights were associated with the second-order analysis of the images (e.g., features computed using the gray-level co-occurrence matrix (GLCM), or the gray-level dependence matrix (GLDM)), whereas only 14 features were related to first-order analysis. In addition, a larger number of “peak” (*n* = 33) versus “early” DCE (*n* = 14) features were present.

**Table 2:** Demographics and baseline features of the included patients

	Value
Gender	
Female	62 (100%)
Mean age (IQR)	49.4 (44.25–53)
MRI exam (*n*)	62
Mean Oncotype Recurrence Score	20.4
Primary pT stage	
T1 (T1a; T1b; T1c)	41
T2	21
T3 and T4	0
Primary pN stage	
N0	31
N1 (N1mi; N1a; N1b)	31
N2 and N3	0
Recurrence score (RS)	
≤ 25	47 (75.8%)
> 25	15 (24.2%)

## Discussion

Our results showed that MRI-based radiomics can predict the risk of recurrence in ER + /HER2 − early breast cancer patients. These findings confirmed the promising preliminary results showing a significant association between radiomics signatures and risk of breast cancer recurrence [38, 40]. For example, Li et al. reported that radiomics features including tumor size and tumor heterogeneity predicted multigene assay recurrence scores [38]. A recent study generated a radiomics signature based on dynamic contrast-enhanced MRI to distinguish between low (recurrence score < 18) and non-low (recurrence score > 18) Oncotype DX risk groups in estrogen receptor (ER)–positive invasive breast cancer [40]. The authors obtained a Rad score based on 10 radiomics features reaching an AUC of 0.759 [40].

Of note, we distinguished low-intermediate risk (recurrence score < 25) and high-risk (recurrence score > 25) patients according to the last American Society of Clinical Oncology (ASCO) clinical practice guideline update. In this regard, the panel of experts referred to the publication of the Trial Assigning Individualized Options for Treatment (TAILORx) evaluating noninferiority of endocrine therapy alone versus chemoendocrine therapy for invasive disease-free survival in women with Oncotype DX scores. Based on informal consensus, the panel recommended that oncologists offer chemoendocrine therapy to patients with recurrence scores of 26 to 30 (9). Only one study adopting this cut-off was recently published in literature for assessing ER + /HER2 − breast cancer patients’ 21-gene RS using a multiparametric MRI-based radiomics model [49]. They obtained an AUC of 0.82 from DCE of the tumor that improved to 0.92 when adding DWI and T2-weighted images. Compared to this study, ours analyzed not only the tumor but also the tissues surrounding the tumor. In detail, the machine learning framework delivered a significant inference on the risk of recurrence when including radiomics features from the tumor and the peritumoral tissues. On the other hand, the standalone combinations of radiomics features did not deliver a significant multivariate inference of the risk. These results are in line with other recent studies, not only focused on breast cancer, showing a potential predictive role of radiomics features extracted from the peritumoral environment [45–48]. For example, Braman et al. investigated the role of MRI-based radiomics signatures to characterize HER2-positive tumor biological factors and estimate tumor response to HER2-targeted neoadjuvant therapy [46]. The authors indicated a classifier performance with an AUC of 0.89 when combining peritumoral and intratumoral features [47]. Other authors investigated the predictive role of DCE-based quantitative features to distinguish molecular subtypes (luminal A/B or basal). They showed that DCE-based features of background parenchymal enhancement were statistically significant in separating luminal A versus nonluminal A cancers and distinguishing basal subtypes [48].

Interestingly, most of the top 5% features derived from 1-mm slice thickness images. This result suggests that high-resolution imaging was a relevant parameter for the prediction performance, and it is in line with Chen et al. that used a cubic spline interpolation algorithm resizing DCE images at 0.9 × 0.9 × 2.2 mm. Most of those features were texture-related, reflecting the degree of heterogeneity in breast tissue.

The role of contrast-enhanced imaging was also relevant in our study. This type of imaging assesses the permeability of blood vessels by using an intravenous contrast agent (gadolinium chelate) that shortens the local T1 time leading to a higher signal on T1-weighted images. The neoplastic neoangiogenesis produces leaky vessels allowing for faster extravasation of contrast agents. This leads to a rapid local enhancement which makes the tumor detectable on post-contrast images [25, 26]. In this regard, 70% of the top 5% features were extracted by contrast-enhanced images obtained at the “peak” of contrast enhancement. These results align with the current state-of-the-art breast MRI that recommends the acquisition of images approximately 60–90 s after the administration of contrast [26]. Although breast MRI without intravenous contrast administration has been proposed as a screening procedure, current techniques, such as DWI, are not sensitive enough to replace DCE-MRI [66, 67].

Our study has some limitations. First of all, it included a relatively low number of patients and lacked a validation cohort. This is due in part to the extraordinary cost of genetic testing that limited the study population size. However, our analysis is set to be a proof-of-concept study, and the nCV implemented in our study minimized the effect driven by the reduced number of samples and overfitting (59). Second, ours is a retrospective single-center study. Further studies, possibly with a prospective design and multicentric, are warranted to confirm our findings and better define the role of radiomics as a predictive biomarker in breast cancer. Third, we only analyzed dynamic contrast-enhanced MRI images, thereby excluding T2-weighted or diffusion-weighted images. Further studies are needed to clarify the potential role of these sequences in tumor recurrence prediction.

## Conclusions

In conclusion, a radiomics-based machine learning approach showed the potential to accurately predict the recurrence risk in early ER + /HER2 − breast cancer patients. Most of the discriminant radiomics features were extracted from high-resolution images obtained at the “peak” of the contrast enhancement. They were mainly related to texture analysis from the tumor and peritumoral environment.

## Data Availability

The datasets generated and/or analyzed during the current study are not publicly available due to the clinical and confidential nature of the material but can be made available from the corresponding author on reasonable request.
